# Oxygen Levels and Immunological Studies

**DOI:** 10.3389/fimmu.2017.00324

**Published:** 2017-03-21

**Authors:** Lauren A. Zenewicz

**Affiliations:** ^1^Department of Microbiology and Immunology, College of Medicine, University of Oklahoma Health Sciences Center, Oklahoma City, OK, USA

**Keywords:** oxygen, hypoxia, T cells differentiation, *in vitro* culture, *in vivo* models

Environmental cues are a major component of directing immune function in health and disease. Cells sense their environment in part through recognition of small molecules such as cytokines, chemokines, and pathogen-associated molecular pattern (PAMP) molecules. This provides immune cells instruction on how to respond to different inflammatory situations. Recent studies in immunometabolism have identified nutrient availability (i.e., glucose or other carbon sources, amino acids, lipids) as an important environmental cue, especially in activated, highly metabolic immune cells (1). Related to nutrients is oxygen, which is critical for most multicellular life as an essential element of several biochemical pathways for the generation of cellular energy. Cells are able to sense oxygen levels and modulate their biosynthetic and transcriptional pathways accordingly.

Cells have two major pathways for generating energy from a carbon source: oxidative phosphorylation or glycolysis. Oxygen is essential for oxidative phosphorylation, the metabolic pathway in which energy is generated through the electron transport chain in mitochondria. In contrast, glycolysis is less fuel efficient but can proceed in the absence of oxygen. In most cells, glycolysis is reserved for when oxygen is limited. However, like tumor cells, activated T cells are able to undergo glycolysis even in the presence of oxygen, a process termed aerobic glycolysis (1). As such, T cells have a distinct relationship with oxygen and modulate their function in response to environmental oxygen levels.

## Oxygen Dynamics in Immune Homeostasis and Inflammation

Oxygen levels vary between 0 and 19% in healthy mammalian tissues. The tissues closest to atmospheric oxygen levels (21.1% or 160 mmHg at sea level) are those of the upper airways (approximately 19%, 150 mmHg) (2). Lymphoid tissues are lower in oxygen; bone marrow is approximately 6.4% (50 mmHg) (2) and the spleen can range from 3 to 4% (25–35 mmHg) (3). The gastrointestinal (GI) tract, which contains upwards of 70–80% of one’s total lymphocytes (4), has an especially dynamic oxygen range (5). The lumen, with its many obligate anaerobic commensal bacteria, is close to 0% oxygen (6). The intestinal tissue, including the lamina propria where many T cells reside, is approximately 7% oxygen (58 mmHg) (2). Immune cells encounter a wide range of oxygen levels as they traffic within the human body (2). T cells begin life in the bone marrow; progenitors migrate to the thymus for development, then to the blood to either circulate through the blood or lymphatic systems or to become a tissue-resident T cell, in such various organs as the lung, skin, brain, or GI tract. A progenitor or mature T cell may be exposed an oxygen concentration between 3 and 19% oxygen. These oxygen levels can be further modulated within the cell’s microenvironment.

Inflammation and environmental oxygen levels are linked; inflammation is often accompanied by hypoxia, and hypoxia itself can cause inflammation (7). In patients, many different inflamed tissues have been shown to have lower than normal oxygen levels. In the GI tract, mice with experimental models of inflammatory bowel disease (IBD) have increased inflammation and decreased oxygen levels in their colonic tissues (8), which corresponds with pathology observed in IBD patients (9). Examining the role of hypoxia in modulating epithelial cell and immune cell responses has been an area of active investigation in the design of new therapeutics for treating IBD (10).

## Hypoxia-Inducible Factor (HIF) Signaling and T Cells

Cells sense and adapt to hypoxia in part through the well-described HIF signaling pathway (11). The activity of this system is regulated by the posttranslational modification and stability of the alpha subunits (HIF-1α or HIF-2α) of the transcription factor complex. In the presence of oxygen, prolyl hydrolyases (PHD) modify the alpha subunits at two prolines, leading to polyubiquitylation and proteasomal degradation. When oxygen levels are low, PHD activity is reduced, which stabilizes the alpha subunits, allowing their translocation into the nucleus, dimer formation with constitutively expressed HIF-1β and binding to coactivators, resulting in transcriptional activation of potentially hundreds of hypoxia-response element-bearing genes. There are also HIF-independent pathways that are induced during hypoxia, including mechanistic target of rapamycin (mTOR) and NF-κB signaling pathways.

Hypoxia-inducible factor signaling regulates many pathways in immune cells, including macrophages, dendritic cells, B cells, and T cells (12, 13). In CD4 T cells, it has positive and negative roles in differentiation of naïve CD4 T cells to different T helper subsets. HIF-1α and low oxygen enhance Th9, Th17, and Th22 differentiation (14–18) but negatively regulate Treg and Th1 differentiation (16, 19). This is in part through interactions of HIF-1α and the critical transcription factors involved in lineage development. In Th17 cells, HIF-1α binds to retinoic acid-related orphan receptor γ (RORγt) and forms a complex on the *Il17a* promoter with p300 thereby enhancing IL-17 production (16). In contrast, HIF-1α binds forkhead box P3, targeting it for proteasome-mediated degradation and reducing Treg differentiation (16). HIF-1α regulates the transcription factor aryl hydrocarbon receptor, promoting type 1 regulatory T cell differentiation (Tr1) (20). In contrast, Th1 differentiation is negatively regulated by HIF-1α, through increased STAT3 signaling (19). The HIF signaling pathway also is important in CD8 T cell biology. HIF, in conjunction with mTOR, is needed for effector function of CD8 T cells (21); however, HIF activation needs to be balanced. In the absence of Von Hippel–Lindau (VHL) protein, a negative regulator of the HIF pathway, HIF accumulation enhances T cell effector function. This allows the CD8 T cells to become refractory to exhaustion, causing immunopathology during a chronic viral infection (22). Further, as VHL deficient CD8 T cells undergo constitutive glycolysis this may preferentially promote T effector memory (23). These examples show some of the numerous HIF-signaling mechanisms affecting the role of oxygen in CD4 and CD8 T cell biology.

Why is environmental oxygen sensing is important for T cells? In addition to the decision between glycolysis and oxidative phosphorylation, I propose this could be another sensor for inflammatory environments. This may be especially true for “sterile” inflammation in the absence of a pathogen, in which the inflammatory milieu lacks PAMPs. My laboratory has proposed that for the dual natured cytokine IL-22, which can be either inflammatory or protective depending on the inflammatory context (24), that oxygen sensing is one tactic cells can use to detect an inflammatory environment and upregulate IL-22 (17). This limits IL-22 to the site of inflammation, where it should be exerting function, instead of in healthy tissue.

## Implications on Research and Experimental Design

A major hurdle in biomedical research is the translation of *in vitro* results to *in vivo* experiments to new drugs and therapies to cure human diseases. Very often *in vivo* results do not agree with the results of initial *in vitro* studies. This is a major hamper to scientific progress and leads some scientists and overseers to question the relevance of *in vitro* studies. Our first thought is to attribute the differences to the *in vitro* cultures for giving false and/or inaccurate results. However, very often the reason is more complicated. I propose oxygen levels may play one role in these differences.

Most *in vitro* and *ex vivo* immunological research studies are performed within a standard laboratory CO_2_ incubator at approximately 17% O_2_. In these experiments, tissues, such as the spleen and lymph nodes, are excised from experimental animals and processed at atmospheric oxygen levels to isolate immune cells. These cells are then cultured in defined media supplemented with fetal bovine sera and incubated in a humidified, 5% CO_2_ incubator at 37°C. Cells may be stimulated with recombinant cytokines or other activating molecules in to order to differentiate and/or activate the cells.

As scientists, we wish to minimize variables in our experiments to increase the probability of reproducibility, defined as the repeatability, robustness, reliability, and generalizability of experiments. Subtle and not so subtle variables in our experiments can make a difference from one set of hands to another and from one laboratory to the next. Differences in atmospheric oxygen levels, between labs at sea level and those at high elevations, may influence results. The concentration of CO_2_ in laboratory incubators may be variable, and these levels should be routinely surveyed for accuracy.

Another issue with increased non-physiological oxygen levels is reduced cell viability. Excess oxygen can be toxic to cells due to increased levels of damaging reactive oxygen species. Enhanced cell survival in low oxygen has long been appreciated by the hematopoietic stem cell (HSC) field (25) and has recently been revisited (26). Mantel et al. showed that isolation of HSCs from bone marrow in a low oxygen environment increased the cell viability and the ability of the cells to transplant into a new host. HSC-derived immune cells may also benefit from such isolation protocols, increasing viability of recovered cells. We should reconsider current incubator designs and include routine experiments to examine if our phenotypes are more apparent/enhanced in lower oxygen conditions, more representative of the host environment.

## Conclusion

As our prioritization of data reproducibility in research continues, it is becoming more important to be cognizant of the numerous environmental factors that shape our immune responses. Immune cells sense oxygen levels and adapt their function to these levels. Oxygen is an important variable that we often fail to coordinate our *in vitro* experiments with physiological levels. As careful and diligent biologists and experimentalists, we need be aware that physiological oxygen conditions and our commonly used methods for *in vitro* cultures do not match. We should, when warranted, evaluate the role of oxygen in our experiments. These deliberations will aid in increasing data reproducibility within and between laboratories, as well as increase translation between *in vitro* and *in vivo* analyses.

## Author Contributions

The author confirms being the sole contributor of this work and approved it for publication.

## Conflict of Interest Statement

The author declares that the research was conducted in the absence of any commercial or financial relationships that could be construed as a potential conflict of interest.
